# Determinants of postnatal spleen tissue regeneration and organogenesis

**DOI:** 10.1038/s41536-018-0039-2

**Published:** 2018-01-16

**Authors:** Jonathan K. H. Tan, Takeshi Watanabe

**Affiliations:** 1 Faculty of Health Sciences and Medicine, Bond University, Gold Coast, QLD 4229 Australia; 2 Laboratory of Immunology, Institute for Frontier Life and Medical Sciences, Kyoto University, Kyoto, 606-8507 Japan; 3 The Tazuke Kofukai Medical Research Institute/Kitano Hospital, Osaka, 530-8480 Japan

## Abstract

The spleen is an organ that filters the blood and is responsible for generating blood-borne immune responses. It is also an organ with a remarkable capacity to regenerate. Techniques for splenic auto-transplantation have emerged to take advantage of this characteristic and rebuild spleen tissue in individuals undergoing splenectomy. While this procedure has been performed for decades, the underlying mechanisms controlling spleen regeneration have remained elusive. Insights into secondary lymphoid organogenesis and the roles of stromal organiser cells and lymphotoxin signalling in lymph node development have helped reveal similar requirements for spleen regeneration. These factors are now considered in the regulation of embryonic and postnatal spleen formation, and in the establishment of mature white pulp and marginal zone compartments which are essential for spleen-mediated immunity. A greater understanding of the cellular and molecular mechanisms which control spleen development will assist in the design of more precise and efficient tissue grafting methods for spleen regeneration on demand. Regeneration of organs which harbour functional white pulp tissue will also offer novel opportunities for effective immunotherapy against cancer as well as infectious diseases.

## Introduction

Spleen is an organ with the innate capacity to regenerate.^1^ Spontaneous tissue regeneration can be observed after cases of splenic trauma, when fragments of ruptured spleen tissue spill into the abdominal cavity and seed the formation of small, spleen-like nodules.^2^ The natural ability for spleen to regenerate has led to the development of spleen auto-transplantation techniques, aimed at preserving or reinstating normal organ function in patients otherwise requiring a total splenectomy. This intervention is essential to maintain blood–borne immunity against encapsulated bacteria such as *Streptococcus pneumoniae*, which is the leading cause of overwhelming post-splenectomy infection (OPSI), and is associated with a 50–70% mortality rate.^3^ Various surgical approaches have been developed to re-implant whole or dissociated spleen fragments into the body,^1^ and these spleen auto-transplantation techniques continue to undergo constant refinement.^4^

Fragments of spleen tissue are typically used for both clinical and experimental spleen auto-transplantation.^5,6^ However, the rationale for transplanting fragmented spleen cubes or slices, the events surrounding graft survival and tissue regeneration, and more precisely the cells controlling de novo spleen formation, are not well defined or understood. Indeed, it is accepted that most graft tissue does not even survive the initial transplantation period, and instead undergoes rapid and almost complete necrosis.^1,7^ When examined in animal models, mostly non-hematopoietic cells have been observed to survive the primary necrotic phase,^7^ raising the possibility that spleen stroma instigates or provides a foundation for new tissue growth. This has been supported by studies transplanting neonatal spleen stromal tissue grafts, prepared by mechanically separating hematopoietic cells from the stromal component of murine spleen tissue.^8^ Transplantation of these grafts have demonstrated that spleen stroma is sufficient to initiate new tissue development.^8^

An underlying contribution of stromal cells to lymphoid tissue organogenesis has been recognised during embryonic development. The formation of lymph nodes and Peyer’s patches is heavily dependent on early interactions between stromal VCAM-1^+^ICAM-1^+^MAdCAM-1^+^ lymphoid tissue organiser (LTo) cells, and hematopoietic CD3^−^CD4^+^IL-7Rα^+^ lymphoid tissue inducer (LTi) cells.^9,10^ Lymphotoxin-α_1_β_2_ (LTα_1_β_2_) is also an essential molecule for lymphoid tissue development, and in the absence of a functional lymphotoxin signalling pathway, both lymph node and Peyer’s patch formation is abolished.^11–13^ Intriguingly, embryonic spleen develops in the absence of lymphotoxin signalling. Lymphoid tissue inducer cells are similarly dispensable, since *RORγ* gene knockout which is critical for LTi development leads to the cessation of embryonic lymph node, but not spleen organogenesis.^14^

To understand these relationships in the context of neonatal spleen regeneration, spleen stromal tissues derived from LTi-deficient RORγt^-/-^ neonatal mice were grafted into wild-type recipients.^8^ Here, the formation of gross spleen tissue was unimpeded, consistent with LTi-independent spleen embryogenesis as previously reported.^14^ However, the transplantation of lymphotoxin-deficient LTα^-/-^ neonatal spleen stromal grafts did not induce tissue regeneration.^8^ This contradicted reports that embryonic spleen develops in the absence of lymphotoxin, as well as transplantation studies involving embryonic day (E)15 LTα^-/-^ spleen grafts which retained the capacity for full tissue development.^15^ Thus, in contrast to spleen development during early embryogenesis, spleen tissue regeneration after birth requires functional lymphotoxin signalling.

## Definition of spleen organiser cells

Specialised subsets of lymphoid tissue organiser cells guide lymph node and Peyer’s patch development.^9,10,16^ Only recently have the equivalent stromal cell subsets controlling neonatal spleen regeneration been functionally defined. Using cell-aggregated grafts constructs, manipulation and transplantation of various CD45^−^ stromal cell subsets enabled the identification of MAdCAM-1^+^CD31 (PECAM-1)^+^ spleen organiser (SPo) cells, which were indispensable for neonatal spleen regeneration.^17^ In common with LTo cells,^18^ organiser cells in spleen expressed high levels of lymphotoxin β receptor (LTβR), as well as ICAM-1 and MAdCAM-1 adhesion molecules. The localisation of MAdCAM-1^+^CD31^+^ cells around the marginal zone of neonatal spleen also supported an organiser cell identity,^17^ indicating a close relationship with CD4^+^CD3^−^ LTi and migratory B220^+^ B cells which simultaneously cluster around the central arteriole during development.^17,19,20^ Notably, MAdCAM-1^+^CD31^+^ cells are also present in human foetal spleen from 14 weeks gestation,^21^ representing a potential spleen organiser cell type in humans.

Rather than a single spleen organiser cell subset controlling tissue formation, a second population of mesenchymal PDGFRβ^+^MAdCAM-1^lo/+^CD31^−^ cells was also found essential for tissue regeneration.^17^ In humans, these cells may correspond to a population of MAdCAM-1^+^CD31^−^ cells that can be detected in foetal spleen from 18 weeks.^21^ At this stage, the precise identity of PDGFRβ^+^MAdCAM-1^lo/+^CD31^−^ cells is unclear and may encompass a range of mesenchymal stromal cell subsets in spleen.^22^ Further examination of the phenotypic profile and spatial localisation will better inform the identity of this cell type, and any potential interactions with MAdCAM-1^+^CD31^+^ organiser cells, LTi, or B cells. Nevertheless, a combination of distinct endothelial and mesenchymal lymphoid tissue organiser subsets is required for lymph node development,^16^ giving precedence for the action of dual organiser populations in spleen tissue regeneration.

## Regulating early and late stages of spleen development

Recent advances have elucidated the cellular components and molecular signalling events driving postnatal spleen regeneration. Yet, how do these findings integrate into a paradigm for secondary lymphoid organogenesis? The regulation of spleen organogenesis is considered distinctive because compared to other secondary lymphoid organs, embryonic spleen develops in the absence of lymphotoxin signalling.^11–14^ However, the spleen is also structurally and functionally unique, exerting dual functions in red blood cell filtration that is carried out in the red pulp, and in adaptive immune responses which are generated in the white pulp.^23^ Contrary to embryonic spleen organogenesis, both MAdCAM-1^+^ marginal zone maturation and white pulp compartmentalisation of T and B lymphocytes occurs postnatally, and both structures are severely compromised in lymphotoxin-deficient mouse models.^11,12^ Therefore, spleen organogenesis can be divided into two separate stages. The initial phase of primitive red pulp formation begins during embryonic spleen development. This occurs independently of lymphotoxin signalling and is instead reliant on the expression of homeobox transcription factors including *Tlx1* and *Pbx1*.^24^ Subsequently, full white pulp and marginal zone formation ensues during postnatal spleen development, and similar to lymph node organogenesis, occurs in a lymphotoxin-dependent manner.^7^

Thus, distinct spleen stromal cell types may exist which independently regulate embryonic and postnatal spleen development. During embryogenesis, specification of spleen mesenchymal cells and formation of splenic anlage commences at E10.5.^24^ Spleen Nkx2-5^+^Islet1^+^ mesenchymal progenitors contribute to early tissue development and give rise to the majority of mesenchymal stromal lineages in spleen including gp38^+^ fibroblastic reticular cells, CD35^+^ follicular dendritic cells and NG2^+^ pericytes.^22^ It has also been shown that lymphoid tissue organiser cells can derive from this pool of multipotent mesenchymal stromal cells.^22^ Since LTo are typically defined as PDGFRβ^+^ cells,^9,25^ early spleen development may in part be regulated by mesenchymal PDGFRβ^+^ organiser cells, but any activity at this stage would not involve lymphotoxin signalling. From E12-E13, LTi or their precursors emerge in foetal spleen.^20^ At E16.5, these cells can be observed surrounding CD31^+^ or VE-Cadherin (CD144)^+^ central arterioles in close proximity to MAdCAM-1^+^VE-Cadherin^+^ endothelial cells,^19,20,26^ thus localising in areas of future white pulp development.^27,28^ At least up to E15, LTi co-localisation with organiser cells in the vicinity of spleen central arterioles occurs independently of lymphotoxin, and these interactions are dispensable for normal spleen development.^14,15^

At a time-point between late embryonic and early postnatal development, lymphotoxin becomes essential for spleen tissue regeneration and organisation of white pulp compartments. Nuclear factor kappa B (NFκB) signalling is critical for lymphoid tissue organiser cell maturation and function,^29^ and in spleen this signalling begins at E17.5.^30^ This may herald the first lymphotoxin-binding events, which trigger NFκB activation through the ligation of LTβR expressed on spleen organiser cells. However, most LTβR-mediated signalling is presumed to occur after birth, when larger numbers of LTi begin to express LTα_1_β_2_.^19^ Even then, LTβR signalling is likely to be augmented by a combination of LTi and mature B cells, which co-localise around central arterioles and functionally express lymphotoxin.^19^ Transgenic animals that selectively delete or express B cell LTα_1_β_2_ further support their involvement in lymphotoxin-mediated white pulp maturation.^31,32^ In addition to white pulp development, lymphotoxin signalling during this specific time window is essential for uptake of neonatal spleen grafts and full tissue regeneration.^8^

## Emergence of the marginal zone and white pulp

Following elevated lymphotoxin cues in the spleen, formation of the marginal zone becomes a critical stage in white pulp formation and ultimately the establishment of immune competence. Between early spleen development and adult marginal zone formation, there is no clear connection surrounding the events leading to marginal zone maturation. Outstanding questions include the origins of the marginal zone, and whether marginal zone reticular cells (MRC) in fact represent adult spleen organiser cells.^27^ In lymph nodes, a positive feedback loop generated by initial LTi engagement leads to the maturation of stromal organiser cells and the upregulation of homoeostatic chemokines such as CXCL13, and VCAM-1, ICAM-1 and MAdCAM-1 adhesion molecules.^33,34^ Consistent with this, upregulation of VCAM-1 and ICAM-1 is observed in stromal cells surrounding the central arteriole in spleen post-birth,^19,20^ at a time when mature B cells influx into the nascent white pulp region.^17,19^ Both of these events do not occur in LTα^-/-^ mice.^19,20^ From this point on, three key reports help shape our understanding of spleen organiser cell maturation and marginal zone formation.

Katakai and colleagues^27^ first presented striking images detailing the expression of MAdCAM-1 in murine spleen from birth to adulthood. At day 0, MAdCAM-1 expression was dispersed throughout spleen tissue, which by day 6 coalesced into foci surrounding central arterioles. The final expansion of characteristic ring-shaped marginal zone structures was observed from 2 weeks of age. In 2009, work by Zindl et al. showed that adult marginal zone sinus-lining vessels, which express both MAdCAM-1 and the arterial endothelial marker ephrinB2,^27,35^ branched directly from the central arteriole. Not only were ephrinB2^+^ cells in the marginal zone a physical continuum of the central arteriole, but cells in this region also expressed markers including CD31, Flk-1 (VEGFR-2) and VE-Cadherin. These reports suggest that after birth, MAdCAM-1^+^ cells in the marginal zone extend directly from and closely encompass the central arteriole, and share the expression of several endothelial cell markers.^27,36^

**Fig. 1:**
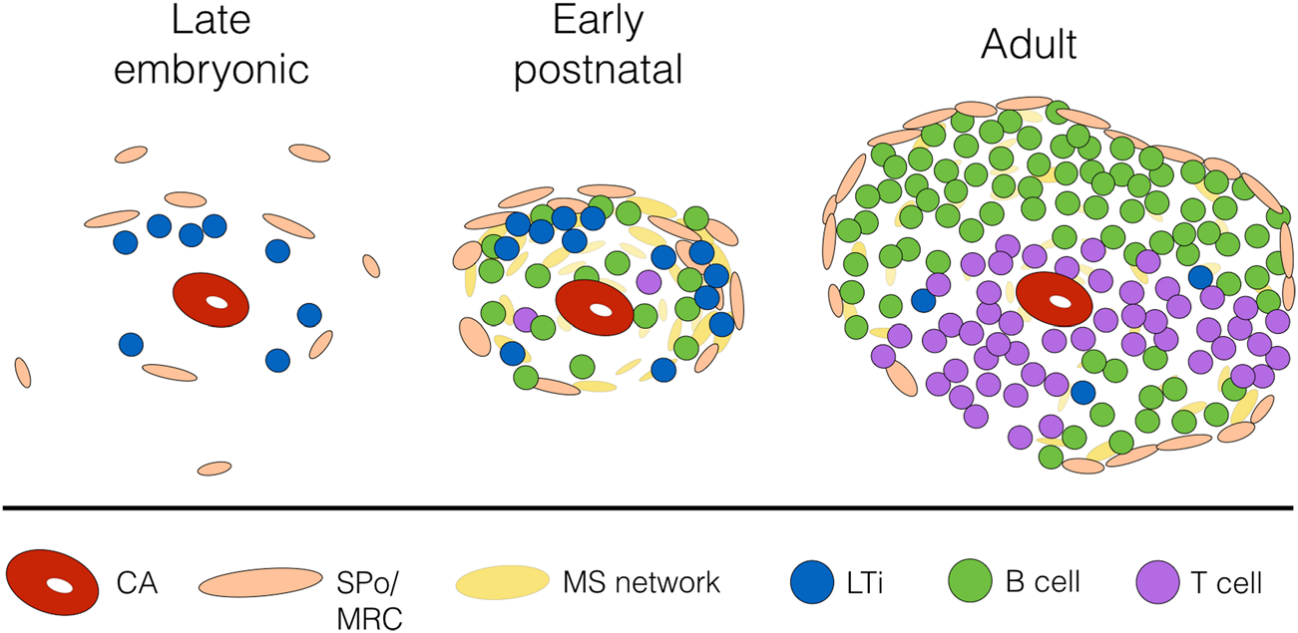
Proposed interactions between spleen organiser cells and LTi/B cells which leads to marginal zone remodelling and adult white pulp formation. During late embryogenesis (E16.5), CD4^+^IL-7Rα^+^ lymphoid tissue inducer (LTi) cells can be observed between the central arteriole (CA) and presumptive MAdCAM-1^+^VE-Cadherin^+^ spleen organiser (SPo) cells.^19^ After birth, LTi continue to co-localise with SPo (MAdCAM-1^+^CD31^+^), which form a primitive marginal zone network surrounding the CD31^+^ central arterioles.^17,20^ This postnatal developmental stage coincides with B cell migration into the spleen,^17^ and both LTi and B cells contribute to lymphotoxin signalling which upregulates MAdCAM-1 expression in the marginal zone, and initiates vascular reorganisation of the Flk-1^+^ephrinB2^+^ marginal sinus (MS) network that extends through the white pulp.^36^ In the adult, MAdCAM-1^+^CD31^+^ cells are localised in the marginal zone and represent mature marginal zone reticular cells (MRC)

The finding that MAdCAM-1^+^CD31^+^ stromal cells are organiser cells functioning in neonatal spleen tissue regeneration^17^ now consolidates these previous reports. Moreover, it supports an ontogenetic relationship between embryonic/neonatal SPo and adult marginal zone reticular cells, which share co-expression of MAdCAM-1 and CD31, and require LTβR signalling to maintain normal architecture.^36^ It is therefore proposed that MAdCAM-1^+^VE-Cadherin^+^/CD31^+^ cells which are distributed around central arterioles shortly before and after birth represent SPo (Fig. 1).^17,19^ Postnatally, these cells are in spatial alignment with LTi and incoming B cells and upon LTα_1_β_2_ ligation, serve as functional spleen organisers. Lymphotoxin signalling during this developmental window is required to enforce MAdCAM-1 upregulation in the marginal zone, which leads to the remodelling of the marginal sinus vascular network and the organisation of adult white pulp structures.

## Summary

Spleen auto-transplantations have been performed clinically for decades, yet the organiser cells controlling tissue regeneration are only starting to be understood. Defining the cellular and molecular events which drive tissue regeneration will be important for addressing limitations in spleen graft functionality, such as the age-related decline in successful spleen auto-transplantations that regenerate full tissue immunoarchitecture.^37^ A reduction in grafting efficiency leads to the development of spleen tissue with poor structural and functional qualities, evidenced by large fibrotic regions and irregular white pulp areas.^38,39^ Similarly, ageing is associated with a general loss of lymphoid tissue organisation and the development of immunosenescence.^40^ Since MAdCAM-1^+^CD31^+^ cells are essential for driving the regeneration of spleen tissue and regulating white pulp organisation, it will be of interest to determine whether numerical or functional changes in this population manifest with age. Answers to these questions may inform better approaches to engineer immune-competent spleen grafts, or may stimulate the development of therapies aimed at directly repairing or rejuvenating aged spleen tissue in situ.
